# Multimodal group-based tele-prehabilitation for cancer patients and caregivers: a pragmatic multicentre hybrid implementation-effectiveness study protocol

**DOI:** 10.3389/fonc.2025.1566489

**Published:** 2025-09-26

**Authors:** Isabelle Doré, Alexia Piché, Corentin Montiel, Sylvie D. Lambert, Chelsia Gillis, Sébastien S. Dufresne, Eléonor Riesco, Pauline Jardel, Michel Pavic, Vanessa Samouëlian, Samuel Dubé, Isabelle Brisson, Danielle Charpentier

**Affiliations:** ^1^ Centre de recherche du Centre hospitalier de l’Université de Montréal, Montréal, QC, Canada; ^2^ École de kinésiologie et des sciences de l’activité physique, Faculté de Médecine, Université de Montréal, Montréal, QC, Canada; ^3^ École de santé publique, Université de Montréal, Montréal, QC, Canada; ^4^ Ingram School of Nursing, Faculty of Medicine and Health Sciences, McGill University, Montréal, QC, Canada; ^5^ St. Mary’s Research Centre, Montréal, QC, Canada; ^6^ School of Dietetics Human Nutrition, Faculty of Agricultural and Environmental Sciences, McGill University, Montréal, QC, Canada; ^7^ Département des sciences de la santé, Université du Québec à Chicoutimi, Chicoutimi, QC, Canada; ^8^ Département de kinanthropologie, Faculté des sciences de l’activité physique, Université de Sherbrooke, Sherbrooke, QC, Canada; ^9^ Centre de recherche sur le vieillissement du Centre intégré universitaire de santé et de services sociaux (CIUSSS) de l’Estrie – Centre hospitalier universitaire de Sherbrooke (CHUS), Sherbrooke, QC, Canada; ^10^ Département de radio-oncologie, Hôpital de Chicoutimi, Chicoutimi, QC, Canada; ^11^ Département d’hémato-oncologie, Centre hospitalier de l’Université de Sherbrooke, Sherbrooke, QC, Canada; ^12^ Institut de recherche sur le cancer de l’Université de Sherbrooke, Sherbrooke, QC, Canada; ^13^ Département d’obstétrique-gynécologie, Faculté de médecine, Université de Montréal, Montréal, QC, Canada; ^14^ Département d’obstétrique-gynécologie, Centre hospitalier de l’Université de Montréal, Montréal, QC, Canada; ^15^ Service de kinésiologie, Fondation Virage, Montréal, QC, Canada; ^16^ Département d’hémato-oncologie, Centre hospitalier de l’Université de Montréal (CHUM), Montréal, QC, Canada; ^17^ Département de médecine, Université de Montréal, Montréal, QC, Canada

**Keywords:** prehabilitation, cancer, pragmatic study, physical health, psychosocial health, implementation science, cancer patients, cancer caregivers

## Abstract

**Background:**

Multimodal prehabilitation can optimize the physical and psychological health of cancer patients, reduce treatment side effects, hospital stay, and accelerate recovery. The support provided by caregivers reduces the demands on the health care system and can be key in the uptake and maintenance of healthy lifestyle behaviours. However, caregivers support comes at a high cost to their own health. Physical activity can help caregivers maintain their health at the level required to successfully perform their vital roles. Our team has designed the first group-based multimodal tele-prehabilitation program targeting both patients and caregivers: coACTIF. This paper presents the protocol of this implementation-effectiveness study.

**Methods:**

This pragmatic, multicentre, hybrid implementation-effectiveness study uses a pre-post-follow-up mixed methods convergent parallel design. The prehabilitation program implementation and effectiveness will be tested in three cities of various sizes in Quebec, Canada. The prehabilitation program includes a virtual supervised group-based exercise program and a web-based educational platform providing learning opportunities and resources on healthy lifestyles and self-management strategies. The study aims to recruit a convenience sample of 100 units (a unit can be a dyad, a patient alone or a caregiver alone). Study participants are French-speaking, adults, preoperative cancer patients and/or their adult caregivers. The implementation and effectiveness are assessed through indicators of the RE-AIM framework: Reach, Effectiveness, Adoption, Implementation and Maintenance. Functional fitness and health outcomes are assessed pre-post intervention and 90-day post-surgery. Interviews with patients, caregivers and health professionals will be conducted to document implementation barriers, facilitators and strategies to facilitate scaling-up of the intervention across various health organisations using the Consolidated Framework for Implementation Research (CFIR).

**Discussion and dissemination:**

This study will provide evidence from various real-world cancer care settings about the implementation and effectiveness of an innovative tele-prehabilitation intervention that aims to rapidly engage cancer patients and caregivers. This intervention has the potential to accelerate and facilitate behaviour change early in the cancer continuum with the objective of optimizing the whole cancer experience and future scaling-up across a variety of cancer care units. Our team will disseminate coACTIF results through reports to stakeholders, scientific manuscripts and presentation at clinical and scientific conferences.

## Introduction

Cancer is a leading cause of morbidity worldwide (1) and mortality in Canada (2). Individuals diagnosed with cancer are at high risk for physical deconditioning (3), malnutrition (4), low quality of life (5), anxiety and depressive symptoms (6), cancer-related fatigue (7) and pain (8). These physical and mental health declines negatively impact disease progression, treatment complications, and survival outcomes that compound to strain healthcare systems. Many caregivers of cancer patients experience fatigue (9), stress (10) and psychological distress (11) that can even surpass levels seen in the patient (12, 13).

Physical activity (PA) before, during and after cancer treatment is a safe and effective intervention that leads to numerous benefits for patients (14–16) in the short and long term (17). Specifically, PA has benefits on physical (e.g., muscle strength, cardiorespiratory fitness (18), pain (19), sleep (19), physical fatigue (20)) as well as mental (e.g., quality of life (21), stress, anxiety and depressive symptoms (21)) health and reduces cancer-specific and all-cause mortality (22). Physical activity in the pretreatment phase (prehabilitation) has been found to build and foster physiological and psychosocial health reserves by optimizing functional, physical, metabolic, and psychological capacities before surgery (23), reduce the incidence and/or severity of symptoms associated with cancer diagnosis and treatments, attenuate physical deconditioning and complications (23), accelerate the recovery after surgery (24) and improve treatment adherence, which in turn reduces the risk of cancer recurrence and mortality (25). Despite cancer-specific physical activity guidelines from national (26) and international (14) organisations recommending regular PA (3 x 30 minutes of moderate-to-vigorous intensity PA/week) and increasing evidence supporting the effectiveness of physical activity to promote health and prevent and reduce physical and mental health symptoms, the vast majority of individuals living with cancer are not active at the level needed to reap the benefits of physical activity.

Supervised PA interventions appear more effective (14, 27) and result in greater benefits on anxiety and depression symptoms, quality of life, and physical function (14, 28) compared to unsupervised. Supervision encourages the development of confidence and a sense of safety and thus, promotes continued PA participation when transferring to an unsupervised setting, such as home (27).

PA interventions delivered in a group context are more effective than individual interventions in enhancing motivation to initiate and maintain behaviour change, which can mostly be attributed to the increased social interactions, social support, and feelings of belonging that happens in a group setting (29). Virtual interventions for cancer patients and caregivers demonstrate feasibility and acceptability evidence (30) to provide efficacious, cost-effective, and tailored PA and educational and self-management support (31). A recent systematic review showed that many cancer patients prefer telehealth PA programs, because of an improved flexibility to accommodate competing commitments e.g., work, family tasks, medical appointments) and removed costs and travel to a medical facility (32) which facilitate intervening within the short window of opportunity between diagnosis and treatment.

Multimodal prehabilitation targets several components, usually, exercise, nutrition, psychological strategies (33), sometimes including respiratory training and motivational techniques to foster sustainable behaviour change (34). These interdisciplinary interventions have greatest impact on patient outcomes compared to unimodal prehabilitation interventions (35). In fact, the multimodal components are necessary as reduced functional capacity cannot always be corrected by PA only, they allow an intervention adapted to the complexity of the cancer and further promotes adherence compared to a unimodal prehabilitation intervention (35).

Multimodal prehabilitation also represents an important strategy to enhance caregivers’ health (36). Cancer not only impacts patients but also places a significant physical and emotional burden on their caregivers. Cancer caregivers (especially females) often experience high levels of fatigue, stress, and anxiety, sometimes exceeding those of the patients themselves, which can hinder their ability to provide effective support (37). Studies suggest that a third of cancer caregivers report clinically significant levels of anxiety 6 months post patient diagnosis and continue to be anxious for up to 5 years (36, 37). Moreover, caregivers’ support reduces the demands on the health care system (38) and positively impacts their loved ones’ illness adjustment. According to a meta-analysis, caregivers who receive psychoeducation, skills training, counselling interventions either independently or in conjunction with the patient, experience reduced caregiver burden, distress and anxiety, and improved coping and physical functioning (39). Despite this, most prehabilitation programs focus solely on patients, neglecting the health and well-being of caregivers, who play a critical role in preparation to the surgery and in improving treatment adherence and recovery outcomes. Including caregivers in prehabilitation could improve their own health and resilience, while also enhancing the overall effectiveness of the intervention for patients. Santa Mina & al. recently proposed a framework highlighting the dynamic and multiphasic potential of prehabilitation that applies to cancer patients, inclusive of caregivers and other relatives (34).

Despite the fact that the diagnosis brings stress and anxiety (40), evidence suggests that cancer diagnosis is a turning point which causes a desire to take control over their life, change their lifestyle habits and prioritize their health and wellbeing (41). The pretreatment phase is a strategic moment to rapidly get patients involved in prehabilitation programs (42) because they have not yet experienced the numerous side effects of cancer treatment and possible complications, which limit their motivation and abilities for behaviour change. Similarly, engaging caregivers early in the cancer continuum might help them get ready for their role and better prepare them to overcome the upcoming challenges they will inevitably face. However, in addition to the common barriers to participation in hospital-based physical activity programs (transportation, parking costs (28), kinesiophobia (43)), specific challenges to engage in interventions in the pretreatment phase include lack of time due to work and numerous medical appointments in preparation for surgery (28).

Although multimodal prehabilitation can provide a multitude of benefits for both cancer patients and caregivers, there is a lack of resources to offer support in adopting and maintaining healthy lifestyles. Thus, based on the most recent scientific evidence, our team designed the first group-based multimodal tele-prehabilitation program, coACTIF (coACTive and InFormed), targeting both patients and caregivers. The 100% virtual format proposed for coACTIF has the potential to reduce inequities in access to supportive care, especially for individuals living outside large cities, in rural and remote regions. Implementability of this group-based multimodal tele-prehabilitation intervention was previously evaluated through a feasibility study in one cancer setting among cancer patients and demonstrated high feasibility, acceptability and fidelity (44). Preliminary efficacy data suggest improvements in both physical and mental health outcomes as well as increased PA behaviour (44). Based on promising results from this implementability study and including recommendations from patient partners and caregivers from the coACTIF co-creation committee we slightly adapted the intervention to better meet the needs of not only patients, but caregivers as well. This study aims to assess coACTIF implementation and effectiveness in real cancer care. This paper presents the protocol of the study.

## Methods

### Study design

We will conduct a pragmatic multicentre hybrid implementation-effectiveness study using a pre-post-follow-up mixed methods (45) convergent parallel design to evaluate coACTIF among patients with cancer and their caregivers. According to the PRagmatic Explanatory Continuum Indicator Summary-2 (PRECIS-2) (46), this study scored 4 (rather pragmatic) or 5 (very pragmatic) on 8 of the 9 domains - eligibility criteria, recruitment, setting, organisation, flexibility-delivery, flexibility-adherence, primary outcome, and primary analysis. Follow-up criteria received a score of 3 (equally pragmatic/explanatory) mainly attributable to data collection tasks specific to the research context (See **Supplementary Material** for PRECIS-2 score wheel). This pragmatic study takes advantage of the real cancer care contexts to emphasize external validity and generalizability to inform future scaling-up across various health organisations.

To support and assess coACTIF implementation and effectiveness, we use the RE-AIM (47, 48) and the updated CFIR (49) frameworks. Used together, these frameworks provide a practical framework for planning and evaluating interventions and inform future scaling-up (RE-AIM) and explains why implementation succeeded or failed (CFIR) by identifying modifiable factors that promote or inhibit adoption, implementation, and maintenance (50). Specifically, the RE-AIM aims at facilitating the translation of scientific advances into practice and include *Reach*, *Effectiveness*, and *Maintenance*– which operate at the individual-level for those who are intended to benefit, and *Adoption*, *Implementation*, and *Maintenance* at the organisational level (47). CFIR is an explanatory model drawing on theories of behaviour change, improvement science, and Diffusion Theory used to document effective implementation by providing tools to identify process and contextual factors from pre-implementation planning to post implementation (49, 50).

### Settings

coACTIF will be delivered in 3 hospitals across 3 regions of Québec: Centre Hospitalier de l’Université de Montréal – CHUM (Montréal, population ~ 1.8M), Centre Hospitalier de l’Université de Sherbrooke – CHUS (Sherbrooke population ~ 180K), and Hôpital de Chicoutimi (Chicoutimi population ~  68K). These regions and hospitals represent a variety of characteristics in terms of geographic location, large urban city vs. small regional city, number of patient consultations per year, and number and type of health professionals. Conducting this project in different regions is essential to reach a broad cross-section of delivery contexts to assess the feasibility of scaling-up the intervention through all of the province of Quebec.

### Participants

Eligibility criteria include: i) being a patient scheduled for cancer-related surgery in at least 2 weeks OR being a caregiver of an eligible patient; ii) being at least 18 years-old; iii) being able to communicate in French; iv) having no contraindications for physical activity. Contraindications are identified using the health screening logic model for exercise preparticipation (51), applied by the kinesiologists on the research team, along with the Physical Activity Readiness Questionnaire (PAR-Q+) (52) completed by participants in REDCap before baseline fitness assessment. If needed, the research team contacts the participant’s physician to obtain medical clearance for exercise. If the participant is not deemed stable enough to engage in moderate-intensity exercise, they are directed to the coACTIF website for information on healthy lifestyle habits, and medical consultation is recommended for further evaluation and clearance. Exclusion criteria include currently receiving neoadjuvant treatments. Although recent evidence suggests that patients receiving neoadjuvant treatments are likely to benefit from prehabilitation intervention (34), the intervention would need to be adapted based on patients’ daily energy and fatigue level. Given coACTIF is a group-based prehabilitation intervention, the level of adaptation that would be required for patients receiving neoadjuvant treatment would be complex and compromise the efficacy for other participants. Patients and caregivers can participate in coACTIF alone or in dyad.

### Sample size

Based on the effect size identified from our feasibility study (44) for our primary outcome, physical fitness (Cohen’s d = 0.82 and 0.49 for 2 min step-test and 30-sec sit to stand test, respectively for pre-post means differences), we need a minimum sample size of 35 participants. We use a conservative effect size of 0.4 with alpha level set at 0.05 and power at 0.80 for Wilcoxon signed-rank test for matched pairs using G-power computation software which suggest a total sample size of 57 participants; considering a retention rate of 60% for post-intervention pre-surgery kinesiology evaluation to measure physical fitness outcomes, we need to enroll 90 participants to assess coACTIF pre-post intervention effectiveness. We did not account for clustering across study sites for sample size computation or analysis since participants are unlikely to share similar clinical or sociodemographic profile according to sites and because the intervention is delivered in groups mixing participants from all three sites. Assuming a 25% drop-out rate at T3, coACTIF will enroll a minimum of 120 individuals. Based on daily consultation rates, we expect to recruit a targeted convenience sample of 100 participant units (a participant unit = a dyad patient-caregiver OR a patient alone OR a caregiver alone) over 12 months (n = 50 (CHUM), n = 30 (CHUS), n = 20 (H. Chicoutimi)) for a maximum possible of 200 individuals. Since we are interested in both overall sample and site-specific indicators of implementation only, a minimal targeted sample of 20 participants per site was selected, based on sample size for pilot study (53).

### Recruitment procedures

A patient-caregiver referral strategy for enrolment was developed to model the conventional clinical referral system. To inform hospital stakeholders (e.g., physicians and surgeons, physician assistants, nurses, and administrative assistants) of the coACTIF program and research project, an advertisement campaign is launched, comprising of presentations during clinical team meetings facilitated by oncologist-managers involved in the project at each site (co-authors VD, MP, PJ), meetings, emails, hospital-TV announcement, poster and flyers in the oncology and surgical waiting rooms. Clinical teams will receive information on the study’s objectives and methodology, including information on how to refer patients, the referral form, and a prehabilitation program handout to review and distribute to patients. Clinicians are advised to introduce the study to patients and caregivers at or near the time of cancer diagnosis and treatment decision-making during medical visits. Potential participants will be directly referred to the research team by email. A research assistant (who is a qualified exercise professional – QEP) will contact referred patients within 48 hours to i) explain the study and program, ii) confirm eligibility, iii) review cancer-related medical history (cancer type, state and metastasis, date of diagnosis and surgery, and past or future treatments) for patients, iv) overview of other health conditions, v) explore physical activity background, vi) schedule baseline fitness assessment. Eligible patients who decline participation in coACTIF intervention or patients eligible for all inclusion criteria except for the minimum two weeks of prehabilitation window (i.e. their surgery is planned in less than two weeks), will be offered to participate in the study for data collection only. Caregivers are recruited through the referred patient where the research assistant asks the patient on their first contact if their caregiver could be interested in participating to the intervention. When they accept, the research assistant collects the contact information of the caregiver and proceeds to the first contact independently. All participants provide online written informed consent.

Immediately following the first call by the research team, the link to access the consent form and the baseline questionnaire on the secured online platform REDCap is emailed to individuals who accept to participate in the study. Once the consent form and the baseline questionnaire are completed, the baseline fitness assessment is performed by the research assistant/QEP to i) review health history and the Physical Activity Readiness Questionnaire for Everyone (PAR-Q+) (54) completed in the baseline questionnaire, ii) take self-reported vital signs ((heart rate and blood pressure measured with a home blood pressure monitor) at rest and anthropometric data (height and weight) when possible, iii) run functional tests, iv) familiarize participants with their individualized exercise program, the structure and functioning of virtual group sessions (including the camera positioning). Each participant receives by email an individualized exercise prescription based on the QEP baseline assessment; they have time to familiarize themselves with the prescribed exercises which will facilitate rapid integration in the exercise group sessions. A maximum of three working days delay is expected from the first call and start of the intervention.

To mimic real-cancer pathway, we use a rolling recruitment strategy; participants integrate exercise sessions following referral and baseline fitness assessment. Thus, the number of participants and the group composition change continuously as new participants enter, and others leave for surgery. The duration of the intervention also varies for each participant depending on the prehabilitation window (referral to surgery time frame).

### Intervention

coACTIF has been developed by a co-creation committee comprising patient and caregiver partners, researchers and health professionals (oncologist, dietitian, nurse, psychologist and QEP). During a total of six co-creation sessions over 2021-2024, the committee i) identified essential components and guided the development of the educational and exercise content of the multimodal prehabilitation intervention, and ii) identified the optimal techno-pedagogical modalities adapted to the intervention, the patients’ and caregivers’ needs, preferences and life context. The coACTIF intervention comprises an exercise component which consists of three 60-min group-based exercise sessions per week for patients and caregivers, and an educational component including evidence-based content accessible on the coACTIF website. The program is free, and no equipment is provided or needed; participants could purchase resistance bands if desired or use body weight or material they already have at home. The exercise component is very similar to the intervention we have pilot tested in our previous study (44); only the structure of the exercise circuits has been revised to maximize aerobic training exercises to follow as closely as possible the cancer and exercise guidelines (14). The educational component, delivered by health professionals on Zoom in the pilot study (44), has been extensively revised; new themes (sleep and motivation) have been added, content has been updated and expanded, and all content have been made accessible through the coACTIF website. We used the Consensus on Exercise Reporting Template (CERT) to describe the intervention (55).

#### Exercise component

Exercise component consists of three supervised 60-min group-based exercise sessions per week for patients and caregivers. Participants from all sites are combined for the intervention; they can choose between morning (9AM to 10AM) or afternoon (3PM to 4PM) group exercise sessions, both offered on Monday, Wednesday and Friday. To facilitate supervision, foster motivation and enhance the sense of belonging to the group, participants cannot alternate morning and afternoon sessions. The program is free, and no equipment is provided or needed; participants can purchase resistance bands if desired or use body weight or material they already have at home. No additional individual home-based exercise is prescribed, but participants are strongly encouraged to remain active on non-session days.

Session supervision is provided by two QEP certified in exercise and cancer who received the 15-hour Thrive Health Certification and a 3-hour coACTIF study specific training. Group-based exercise sessions are delivered using synchronous videoconferencing technology with Zoom software (Zoom Video Communications, San Jose, CA); one QEP leads the session and the other monitor for safety and provide individual support or specific indication when needed (moderator). QEPs supervise exercise sessions from their organisation’s facilities or home office, using the Zoom platform, a computer with a large or dual-screen monitor, a wireless microphone, and a high-quality camera. Participants join sessions from home via Zoom, using a camera- and microphone-equipped device (e.g., laptop or tablet). Participants are asked to adjust their camera so the QEPs can always see their entire body.

The 60-min session includes the following: i) a 10-min warm-up (including mobility exercises and progressively increasing the intensity with light intensity cardio exercises); ii) a main session with three cardio stations of 5-min bouts and two resistance circuit stations targeting major muscle groups and surgery specific muscles; and iii) a 5-min cool down (including flexibility and breathing exercises). A surgery-specific functional exercise is included to optimize pre-surgical preparation, targeting either mobility or strength. For example, a shoulder mobility exercise will be prescribed for a participant awaiting a mastectomy if there is an asymmetry in shoulder range of motion in order to facilitate the return to full range prior to surgery. See **Supplementary Material** for detailed structure of the exercise session including type of exercise, number of sets, repetitions and duration. Effort intensity is monitored with the 10-point Borg Rating of Perceived Exertion (RPE) scale (56), targeting 3 to 6 (moderate intensity) to ensure the safety of participants at home. Progression is ensured by increasing the level of difficulty of the exercise when the previously prescribed activity no longer reach the target intensity or becomes too easy. The session structure is standardized for all participants, but the prescribed exercises are individualized. Specifically, participants are classified as beginner, intermediate, or advanced based on the QEP’s clinical judgment which considers baseline fitness assessment tests, physical activity experience and medical history of the participant. This classification informs the exercise prescription and the tailored guidance provided by the QEPs during exercise sessions.

The real-time interactive supervision and support provided by QEPs during each online session are key to ensure participant safety, appropriate exercise execution, personalized adaptation and foster motivation. Supervision includes the following key components: i) monitoring safety: by observing participants throughout the session to identify signs of fatigue, and intervening as needed to prevent injury, ii) providing real-time feedback, guiding participants on proper technique, posture, and breathing during exercises, iii) adapting exercises if and when necessary by modifying intensity, range of motion, or type of exercise based on participants’ individual functional abilities, symptoms, or medical limitations, iv) fostering engagement: encouraging participation, motivation, and interaction among group members and v) ensuring fidelity to the session content according to the standardized coACTIF protocol, ensuring consistency across sites and sessions.

No more than 12 participants per group (AM or PM) will be included to ensure adequate monitoring for safety. Adherence to exercise prescription is not measured, but attendance is reported for every session. QEPs use a tracking and attendance sheet which includes key participant information: i) phone number, ii) home address, iii) emergency contact, iv) complete health history and QEP baseline fitness assessment note, v) specific consideration for physical activity, vi) date for surgery. This sheet is always accessible during group sessions to provide rapid assistance in case of an adverse event. Moreover, participants inform QEPs if they are alone and at home before every session. In case of any adverse event during or after exercise sessions, a report is filled by the research team to document the type and number of adverse events.

Fidelity checks are carried out by a research coordinator who is a QEP to ensure consistency, conformity and safety in the delivery of the exercise intervention across QEP and over time. A random 10% of exercise sessions are observed and reviewed by the research coordinator using a standardised fidelity checklist (57) (See **Supplementary Material**). Feedback to improve delivery is provided to the QEPs.

#### Educational component

coACTIF intervention also includes a web-based educational component co-constructed with patient and caregiver partners, accessible through the coACTIF website (coactif.ca) to help participants adopt and maintain healthy lifestyle habits. coACTIF website proposes 18 learning modules grouped under 5 themes: physical activity, nutrition, well-being, sleep, motivation and self-management. Based on previous work from TEMPO program (58), self-management techniques, using self-monitoring, goal setting, information seeking, decision-making, and action planning to foster heathy lifestyle habits adoption and maintenance and build confidence for enacting those skills are integrated in various modules to support behaviour changes (59). All modules include general and specific information addressing patients and caregivers’ specific needs. Each learning module includes i) a short 5-8-min video presentation, ii) a printable detailed document with all content delivered through the video, iii) quiz with feedback, and iv) resources for additional information and support. Every Friday, participants receive an email from the research team reminding them to consult the coACTIF website educational modules based on their needs and interest. Main themes and specific modules content details are provided in **Table 1**.

**Table 1:** Educational themes and modules.

THEME	MODULE TITLE
PHYSICAL ACTIVITY	
1	Being active from the diagnosis to rehabilitation
2	Being active in daily life & avoiding sedentarity
3	Warm-up, cool down and mobility
4	Cardiovascular training
5	Resistance training
NUTRITION	
1	Preparing for surgery with nutrition
2	Managing cancer-related symptoms and side effects through nutrition
3	Nutrition from a caregiver’s perspective
4	Myths: nutrition and cancer
WELL-BEING	
1	Taking care of your physical and overall health
2	Understanding emotions and anticipating reactions
3	Communicating, surrounding yourself and setting limits
SLEEP	
1	Why sleep is important?
2	Sleep perturbations
3	Sleep management strategies
MOTIVATION	
1	Taking action
2	Setting goals
3	Maintaining motivation

### Data collection

Data collection for coACTIF intervention participants includes the following: i) a *study logbook* to record information on recruitment, participation, and research observations during program delivery, collected by the research assistants and the QEPs involved in exercise sessions; ii) *fitness assessments*, conducted virtually by a QEP at baseline pre-intervention (T1), post-intervention pre-surgery (T2) and follow-up 90-day post-surgery (T3) to assess physical fitness; iii) *self-report questionnaires* at T1, T2, T3 to document the sociodemographics, clinical profile, health outcomes and intervention satisfaction; iv) *self-report weekly questionnaire* during the intervention to assess coactif.ca education platform consultation frequency, content satisfaction and utility; vi) *data extracted from patient medical record* 30 days after surgery; *interviews with patients and caregivers* (n = 15 participant units, diversity of dyads, patients or caregivers alone from the three settings) select by the research team based on their interest in participating in this data collection and diversity in terms of sex, age, cancer type, at the end of program delivery (T4); vii) *interviews with stakeholders* (n = 10, diversity of health professionals and managers from the three settings) at the end of program delivery (T4).

**Figure 1:**
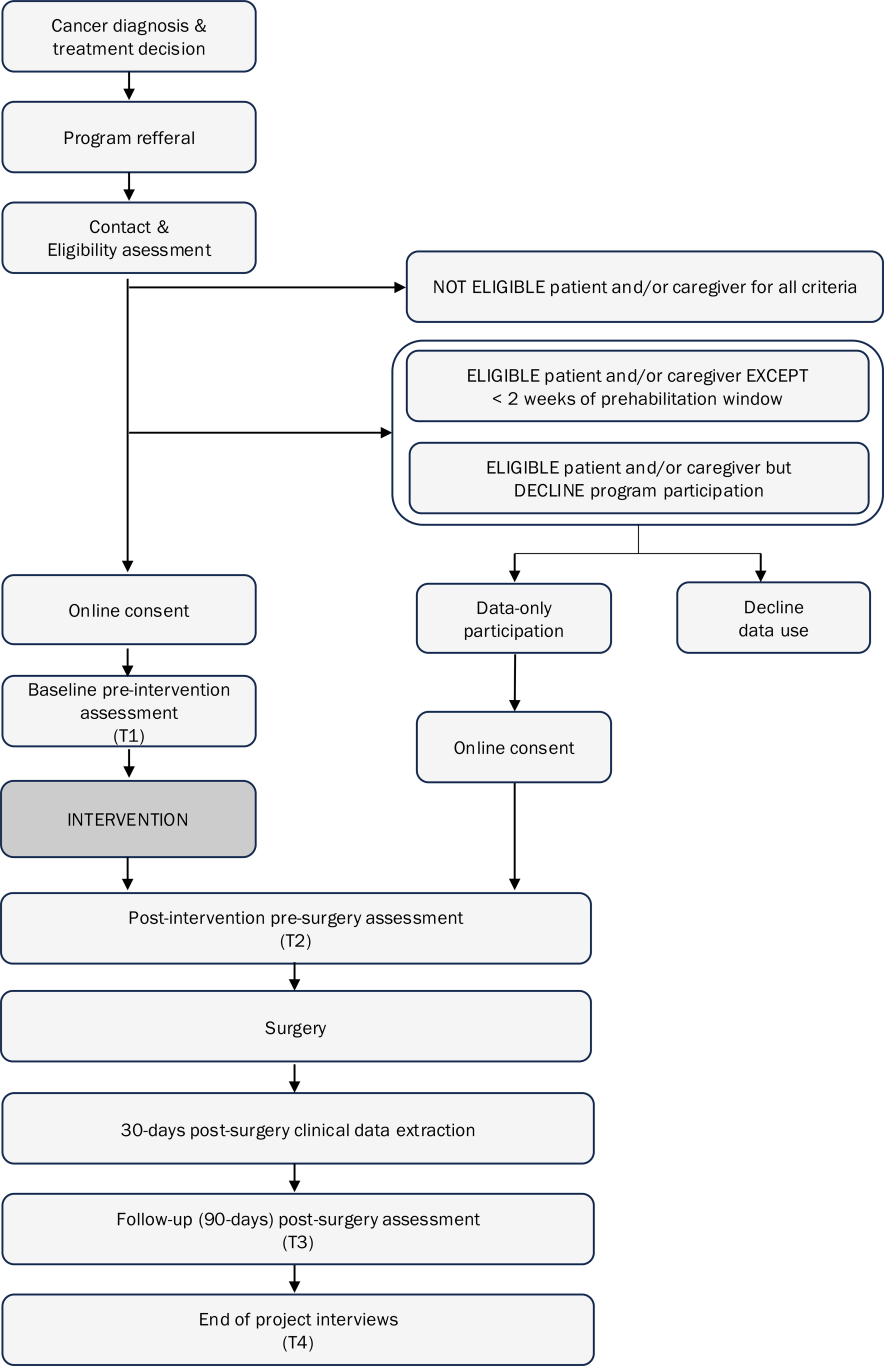
Recruitment and data collection flow chart.

**Figure 2:**
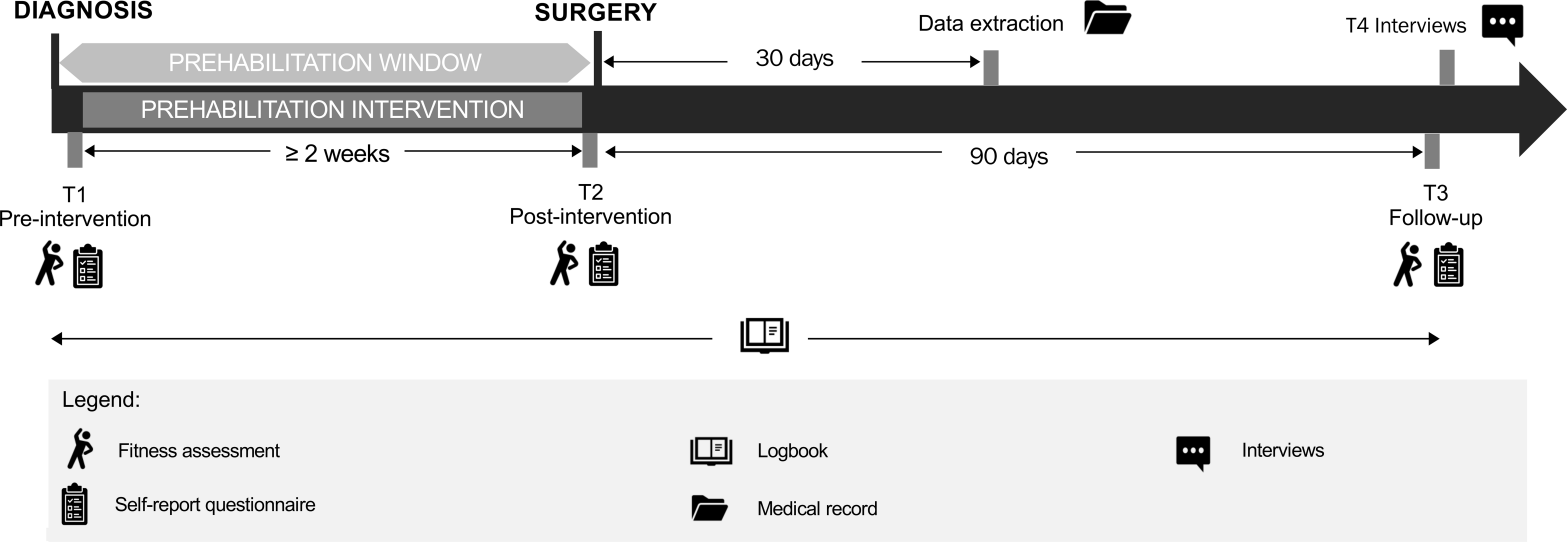
Study timeline.

Data collection for coACTIF data-only participants includes *data extracted from patient medical record* 30 days after surgery and, *fitness assessments* and *self-report questionnaires* pre-surgery (T2), if time allows before surgery, and 90-day post-surgery (T3). See **Figure 1** for study recruitment and data collection flowchart. See **Figure 2** for study timeline.

To ensure standardization of data collection across the three sites, all quantitative data (study logbook, baseline assessment and questionnaires) are collected by the research team using the secure online platform REDCap (REDCap 14.5.24, © 2024 Vanderbilt University) and data are stored on the research center’s local server to ensure security. Semi-structured interviews are audio-recorded on Zoom and transcribed verbatim.

### Nutrition screening and referral

Since malnutrition and sarcopenia are independent predictors of adverse postoperative outcomes (60, 61) and associated with worse physical functioning (62, 63), all participants will complete the Canadian Nutrition Screening Tool (CNST) (64) at T1 to identify those at risk of malnutrition. Participants who respond yes for both items of the CNST (i.e. *Have you lost weight in the past 6 months without trying to lose this weight?* and *Have you been eating less than usual for more than a week*)? are considered at risk. Given all measurements are conducted virtually, sarcopenia is assessed with the 5 items of the SARC-F, which has good reliability but low to moderate sensitivity (65), and the SARC-CalF (calf circumference) (66) which seem to improve specificity but not sensibility of sarcopenia screening (67). At risk participants are directed to the hospital-oncology dietitian on site for a complete assessment and malnutrition diagnosis. The dietitian will provide these patients with additional personalized tips to improve nutritional status. In addition, they are instructed to consult the Nutrition content of coACTIF educational platform, based on MacMillan cancer support guidelines (68).

### Implementation and effectiveness indicators

As mentioned previously, the RE-AIM and CFIR framework are used to evaluate coACTIF. Indicators have been organized according to *Reach*, *Effectiveness*, and *Maintenance (behaviour)* indicators that operate at the individual-level, and *Adoption*, *Implementation*, and *Maintenance (intervention)* indicators at the organisational level. A summary of all indicators, measures, data collection tools and time of data collection are presented in **Table 2** and detailed below. The CFIR framework will be applied to identify contextual factors influencing implementation success or failure. Specifically, CFIR domains will be explored as follows: *Outer setting* - Barriers and facilitators related to geographic accessibility, patient-caregiver needs, and external policies will be identified through semi-structured interviews with participants and stakeholders; *Inner Setting* - Organisational capacity, communication, and staff engagement will be assessed via logbooks and interviews with healthcare providers and managers; *Characteristics of Individuals* - Perceptions, attitudes, and engagement of participants and healthcare professionals will be explored using participant-reported questionnaires and qualitative data from interviews; *Intervention Characteristics -* Factors such as intervention complexity, adaptability, and perceived value will be analyzed through fidelity checks, participant feedback, and QEP group interviews; and *Process -* Key implementation steps, such as stakeholder engagement and intervention adaptation, will be examined through documentation and interviews.

**Table 2:** coACTIF Indicators, measures, data collection modality, and time.

Indicators	Measures and tools	Modality	T1	T2	T3	T4
REACH						
Referral						
Indirect-HCP referral	#, professional type	Logbook	x			
Direct-HCP referral	#, professional type	Logbook	x			
Self-referral	#, source of information	Logbook	x			
Enrolment						
Participants enrolled	#, Characteristics	Logbook	x			
Participants not enrolled	#, Characteristics, Reasons for study refusal	Logbook	x			
Barriers						
Internet accessibility		Logbook	x			
Others		Logbook	x			
EFFECTIVENESS						
Fitness outcome						
Aerobic fitness	2-min step test	Fitness assessment	x	x	x	
Lower body strength	30-sec sit-to-stand test	Fitness assessment	x	x	x	
Lower body endurance	60-sec sit-to-stand test	Fitness assessment	x	x	x	
Balance	Single-leg balance test	Fitness assessment	x	x	x	
Behaviour and Health outcomes						
Malnutrition	Canadian Nutrition Screening Tool (CNST)	Self-report questionnaire	x	x	x	
Physical activity practice	International Physical Activity Questionnaire (IPAQ)	Self-report questionnaire	x	x	x	
Kinesiophobia	1 item of the Tampa Kinesiophobia Scale (KTS)	Self-report questionnaire	x	x	x	
Stress	Perceived Stress Scale (PSS)	Self-report questionnaire	x	x	x	
Anxiety-depressive symptoms	Hospital Anxiety and Depression Scale (HADS)	Self-report questionnaire	x	x	x	
Sleep quality	Pittsburgh Sleep Quality Index (PSQI)	Self-report questionnaire	x	x	x	
Health-related quality of life	Functional Assessment of Cancer Therapy – General (FACT-G)	Self-report questionnaire	x	x	x	
Fatigue	Functional Assessment of Chronic Illness Therapy-Fatigue (FACIT-F)	Self-report questionnaire	x	x	x	
Self-management skills	Partners in Health	Self-report questionnaire	x	x	x	
Medical outcomes						
Hospital length of stay	*Hospital length of stay (# of nights)	Medical record			x	
Complications	*30-days postoperative complications	Medical record			x	
Readmission	*30-day postoperative readmission	Medical record			x	
ADOPTION						
Characteristics of stakeholders involves						
HCP involve in referral	#, professional type	Logbook	x			
Resources for referral	Type	Logbook	x			
QEP trained for program delivery	#, professional type	Logbook	x			
IMPLEMENTATION						
Feasibility						
Eligible referral rate	Eligible referrals/total referral	Logbook	x			
Eligible referral speed	Eligible referrals/month	Logbook	x			
Recruitment rate	Participants recruited/eligible referrals	Logbook	x			
Prehabilitation window	Time from referral – to date of surgery	Logbook	x			
Prehabilitation duration	Time from T1 – to T2 assessment	Logbook	x			
Study retention	Participants with T3 assessment completed/total participants	Logbook				x
Barriers & facilitators	Obstacles and strategies for participation	Participant interview				x
Barriers & facilitators	Obstacles and strategies for referral or delivery	HCP & QEP interview				x
Safety						
Perceived safety	Perception of safety during exercise session	Self-report questionnaire		x		
Falls	# of falls during exercise sessions	Self-report questionnaire		x		
Adverse event	Tracking and reporting by QEP	Logbook	x			
Acceptability - Exercise session						
Attendance	Exercise sessions attended/possible	Logbook		x		
Satisfaction	Duration, frequency, virtual format	Self-report questionnaire		x		
Sense of belonging	Relatedness to Others in Physical Activity Scale	Self-report questionnaire		x		
Acceptability - Educational content						
Adherence	% Educational content consulted as prescribed	Weekly questionnaire		x		
Appreciation and utility	Scored on a 10-point scale for content consulted	Weekly questionnaire		x		
Acceptability - Overall intervention						
Utility for lifestyle management	Questionnaire developed for this study	Self-report questionnaire		x		
Fidelity						
Exercise sessions	Fidelity check: Session conformity + consistency	Logbook	x			
Overall fidelity	QEP training, experience, fidelity and adaptation in delivery	QEP interview				x
Program costs						
Costs	Delivery and administrative support costs	Logbook				x
MAINTENANCE						
Physical activity practice	International Physical Activity Questionnaire (IPAQ)	Self-report questionnaire			x	
Participation in rehabilitation	Subscription to a rehabilitation program	Self-report questionnaire			x	
Organisational sustainability	Region offers the program after the study	Logbook				x
PARTICIPANTS CHARACTERISTICS						
Clinical data	Chronic conditions and comorbidities, *cancer site, *stage at diagnosis, *date of diagnosis, *date of surgery, *type of surgery	Self-report questionnaire	x			
Sociodemographic data	Sex, age, country of birth, household composition, education level, employment, family income, patient-caregiver relationship	Self-report questionnaire	x			

HCP, Health care provider; QEP, Qualified Exercise professionals.

*Patient only.

### Reach

Reach indicators first include *referral* measures: we document the type of referral categorized as “Direct referral” – when the research team received a referral directly from the health care provider (HCP), “Indirect referral” – when the participant contact the research team after receiving information or recommendations from the HCP and “self-referral” when the participant contact the research team without any information provided by a HCP (e.g., participant heard about coACTIF through word of mouth or hospital information sessions, saw a poster or a pamphlet in the hospital waiting room). Number of each type of referral is collected; for direct and indirect referral from a HCP, we ask about the type of professional who referred and for self-referral we document the source of referral. We also assess reach through *enrolment* (number and characteristics of participants enrolled and not enrolled and reason for study refusal) measures as well as *barriers* specific to reach (e.g. internet accessibility, ease of use with technology, etc.). All information is collected by the research coordinator in the study logbook.

### Effectiveness

Effectiveness of the intervention is assessed through outcomes of fitness, behaviours, physical and mental health symptoms and medical care measures. These data are collected through i) fitness assessments, ii) self-reported questionnaires and iii) data extracted from medical records.

#### Physical function

Physical function is measured during baseline assessment through measures of *aerobic fitness* with the 2-min step test (69)—the number of steps at a targeted height (mid-point between patella and iliac crest) in 2 min, *lower body strength* using the 30-sec sit-to-stand test (70)— the number of full stands in 30-s starting in seated position with arms crossed on the chest, *lower body endurance* using the 60-sec sit-to-stand test (71) and *balance* using the single-leg balance test (72). An increase in repetitions or duration indicates favorable change. These tests have demonstrated acceptable reliability and validity in a remote setting (73).

#### Behavioural and health outcomes

Outcomes measured in the self-reported questionnaire include *weekly physical activity* using the International Physical Activity Questionnaire (IPAQ) (74) which provide measure of frequency and duration of light (walking), moderate and vigorous intensity physical activity and of sedentary activities. *Kinesiophobia* is assessed using the first item of the Tampa Kinesiophobia Scale (TSK) (75): “I am afraid that I might make my symptoms worse if I exercise”*. Stress* is assessed using the Perceived Stress Scale (PSS) (76) which comprises 10 items asking about feelings and thoughts through perceived helplessness (six items) and perceived self-efficacy (four items). PSS had acceptable psychometric properties in various populations, including cancer (77). *Anxiety and depressive symptoms* are measured using the Hospital Anxiety and Depression Scale (HADS) (78) which comprises seven items assessing anxiety symptoms and seven items assessing depressive symptoms. The psychometric properties of HADS have been assessed in various populations and the French–Canadian version shows good reliability and validity (79). *Sleep* is measured using the Pittsburg sleep quality index (PSQI) (80) which comprises 19 items assessing subjective sleep quality, sleep, latency, sleep duration, habitual sleep efficiency, sleep disturbances, use of sleeping medication, and daytime dysfunction. Psychometric evaluation of the PSQI supports its internal consistency and construct validity in clinical and non-clinical population (81) and in cancer patients specifically (82). Health-related quality of life is measured using the Functional Assessment of Cancer Therapy-General (FACT – G) (83) for cancer patients and the adapted version for caregivers. FACT-G is a 27-item measure including for subscales assessing physical, functional, social/family (and emotional well-being, validated among cancer patients in various languages including French (84); the adapted version for caregivers has been validated in English among family cancer caregivers (85). Using French items of the FACT-G for patients, our team developed the FACT-G for caregivers into French which only required only minor translation. *Fatigue* is assessed with the Functional Assessment of Chronic Illness Therapy-Fatigue (FACIT-F) (86) which cover the full range of the fatigue spectrum; the measure has been translated in various languages, including in French (87) and among various clinical populations (88) and in general population (89). Self-management skills are measured with the Partners in Health (PIH) tool, comprising 12 items (90), adapted and validated in French (91) for patients with chronic conditions. There is currently no PIH adapted to caregivers; consequently, our team undertook the adaption of the PIH for caregivers following a method used previously for similar questionnaires (91, 92) which include the following steps: 1) adaptation of each item by four independent researchers or graduate students with relevant expertise (co-authors (ID, AP, CM, SL), 2) committee evaluation to clarify inconsistencies and reach consensus and 3) pretest among a sample of 10 individuals.

#### Medical outcomes

Medical outcomes will be extracted from patient medical records and include hospital length of stay following surgery, 30-days postoperative complications and 30-days readmission post-surgery.

### Adoption

To assess adoption of coACTIF intervention in each site we will document in the logbook the number and type of stakeholders involved in referral and delivery (e.g. managers, clinical research personal, HCP), resources that are being used for coACTIF exercise program promotion and referral and educational platform dissemination as well as the number of QEP trained to deliver coACTIF exercise sessions.

### Implementation

#### Feasibility

Feasibility (i.e., the extent to which the intervention can be successfully carried out in a real care context) will be measured using *eligible referral rate* [eligible participants/total referrals] and *eligible referral* sp*eed* [average number of eligible referrals/month], *recruitment rate* [recruited participants/eligible referrals], *prehabilitation window* [time since referral to surgery] and *prehabilitation duration* [time since T1 to surgery], *study retention* [participants who complete T3 assessment-questionnaires/total participants]. All this information is collected by the research coordinator in the logbook. *Obstacles encountered and strategies* used to overcome these obstacles for *program participation* will be documented through semi-structured individual interviews with participants and for *referral and delivery* will be discussed with HCP and QEP also using semi-structured individual interviews.

#### Safety

Safety will be self-reported by participants in the T2 self-report questionnaire through perceived safety (1 question) and number of falls (one question) during exercise sessions. *Adverse events* are registered by the QEP during exercise sessions via the Common Terminology Criteria for Adverse Events V5.0 (93).

#### Acceptability

Acceptability (i.e., the extent to which the intervention is considered appropriate, satisfactory, useful or attractive by program beneficiaries) of the exercise session, educational content and overall coACTIF intervention will be assessed. Exercise session acceptability indicators are self-reported in the T2 questionnaire and include *exercise session attendanc*e [number of exercise sessions completed/number of possible sessions—based on the participant specific prehabilitation duration] collected by the research coordinator in the study logbook, s*atisfaction of exercise session structure* (frequency, schedule, duration, intensity, difficulty level, progression), s*atisfaction of telehealth format*, s*atisfaction of group format* and *satisfaction of the supervision by the QEP* as well as *sense of belonging* to the exercise session group assessed with the Relatedness to Others in Physical Activity Scale (ROPAS), a valid and reliable tool (94). Educational content acceptability is assessed through *educational content adherence* [% educational modules consulted among all content available] and *appreciation and utility* of specific content consulted both self-reported in the weekly questionnaires. *Overall intervention satisfaction* is measured through a questionnaire developed by our team specifically for this study to document *utility for lifestyle management* based on the Nutrition and Dietetic Patient Outcomes Questionnaire - Adult (NDPOQ-A) (95) in the T2 self-reported questionnaire.

In addition, interviews will be conducted with a purposive sample of participants, HCPs and QEPs to provide a complementary understanding of the program implementation. Sampling of participants includes considerations of unit composition (dyad, patient only, participant only), location, participant age, cancer diagnosis, gender and physical activity levels at baseline. For managers and HCPs, sampling considers location, role and years of experience. All QEP involved in program delivery will be invited to participate in a group interview. This purposive sampling will ensure diversity of views across program participants, managers, HCPs and QEPs. Semi-structured individual interviews will be conducted at the end of the 12-month intervention delivery (T4) to identify barriers and facilitators to program participation and program perceived effectiveness and utility (participants) and program promotion and referral delivery (managers and HCP) and suggestions for improvement. Individual interviews will be conducted by phone or videoconference by two research assistants and will last ~30–45 minutes. A 60-min semi-structured group interview with the QEPs involved (n = 5-7) in the exercise intervention delivery will be conducted to gather information on challenges and facilitators to program delivery and fidelity. All interviews will be audio-recorded and transcribed verbatim. Qualitative data will provide a complementary understanding of the program implementation from the diverse perspectives of participants, managers, HCP and QEP to supplement, enrich and potentially explain results emerging from quantitative implementation and outcomes data.

#### Fidelity

Fidelity (i.e., the extent to which the intervention is deployed as planned and adaptations to improve the intervention facilitate its implementation) will be measured using random unannounced monitoring fidelity checks conducted by the research coordinator during 10% of the exercise sessions over the 12-month delivery of the program. The research coordinator will observe the session and collect information in the study logbook to assess i) *session conformity* (how well the intervention parameters are delivered as planned) by documenting the structure of the group sessions (time allowed, warm up, circuits, and cool down), and ii) *session consistency* (type and volume of adaptations required to ensure the appropriate intervention delivery) over time and between QEPs (96). At T4, supplementary fidelity data will be collected in a semi-structured group interview with the QEP involved in program delivery to complement logbook information and the random check including QEP training, work experience, conformity of the intervention, and consistency in delivery over time and between QEP.

#### Program costs

Overall program costs will be estimated based on training, personnel/administrative support, site-specific costs related to program promotion, printed material, etc.

### Maintenance

Maintenance is assessed at the individual level through physical activity behaviour and subscription to an exercise rehabilitation program self-reported by the participants 90 days post-surgery at T3 questionnaire. Maintenance at the organisational level refers to whether the region/site was able to maintain the program delivery after the 12-month period planned for the study.

### Participants characteristics

Sociodemographic and clinical data will also be collected through the self-reported questionnaire at T1 to describe participants and conduct subgroup analysis. Sociodemographic characteristics include gender, age, country of birth, ethnicity, aboriginal status, postal code, household composition, education, employment status, household income and patient-caregiver relationship when applicable. Clinical characteristics include cancer site* date of diagnosis*, date of surgery*, type of treatments planned*- including surgery, neoadjuvant and adjuvant treatment, risk factors for cardiovascular disease, other diseases, and BMI (data with a * will be collected only among patients since they do not apply to caregivers).

### Analysis

Descriptive statistics will be performed for implementation and effectiveness outcomes (frequency, percentage, range, median, mean, standard deviation). Paired sample Student’s *t*-tests (or Wilcoxon signed-ranked test for non-normally distributed data) will be used to analyze pre-post (T1-T2) intervention changes in participant-related outcomes. Cohen’s *d* effect sizes will be computed. Reach, adoption, medical outcomes, feasibility, program costs and organisational maintenance will be compared according to hospital site. For participants who do not complete a fitness assessment (T1, T2, or T3), fitness outcome values will be recorded as missing by the kinesiologist in the REDCap evaluation chart. Missing values for variables and outcomes collected via self-reported questionnaires—whether due to incomplete responses or unanswered specific items—will be automatically recorded as missing in REDCap. For the analysis, a complete case analysis will be conducted first. Sensitivity analyses using multiple imputation by chained equations (76) will be performed to replace missing values for individuals not lost to follow-up. Participant-related outcomes, safety, and acceptability will be compared according to prehabilitation duration (time from T1 to T2 assessment), exercise session attendance (number of sessions attended/total possible according to prehabilitation duration), treatment received, and unit composition (dyad, patient alone, or caregiver alone) to document potential differences. Physical fitness and medical outcomes will be compared between coACTIF intervention participants and data-only participants. All analysis will be conducted using R (4.4.1). Interview qualitative data will be analyzed using conventional content analysis (97) Context-specific factors will be identified through a mostly inductive coding procedure that will be guided by the overarching constructs of the CFIR. Data will be coded and categorized by two experienced research assistants and differences will be discussed to reach consensus and create a common codebook. Data will be coded with MaxQDA (22.0.0).

## Discussion

The increase in cancer diagnosis, as well as recent major public health challenges such as the COVID-19 pandemic, have restricted and delayed access not only to cancer surgery and treatment (98, 99) but also to supportive care (100) aiming at optimizing physical and mental health of patients. In this exceptional context, many oncology supportive care teams had to rapidly adapt to transfer in person services into telehealth interventions (101, 102). Rather than being a limitation, the telehealth modality became a key solution for prehabilitation, by facilitating rapid enrollment of patients recently diagnosed with cancer through virtual fitness assessment and intervention within the short window of opportunity between diagnosis and surgery (103). Digital access inequity can be a barrier to participation in virtual health interventions, particularly among individuals with limited digital literacy, restricted access to reliable internet or devices, or discomfort with online platforms. To address this challenge, coACTIF implements targeted strategies, including personalized telephone support, in which trained staff provide step-by-step guidance to participants for setting up, navigating, and using the Zoom platform. In addition, a written guide with images illustrating Zoom functionalities and connection procedures is provided to all participants. Many studies, including those in the specific context of the COVID-19 pandemic, have also shed light on the critical roles of caregivers (104–106). The emotional and social support provided by caregivers reduces the demands on the health care system, positively impacts their loved ones’ illness adjustment and can be key in the uptake and maintenance of PA. Regular PA also represents an important self-management strategy to help caregivers maintain their health at the level required to successfully perform their vital roles (36). However, despite the increased interest in physical activity programs to optimize both physical and psychosocial health of cancer patients and the evidence suggesting acceptability and feasibility of virtual intervention, most telehealth PA interventions for cancer patients to date i) are limited to rehabilitation, ii) target only PA (i.e., unimodal) and ii) are individual in nature, thus limiting social interactions likely to promote sense of belonging and social support which improves motivation for participation and iv) only few include caregivers. This study will provide evidence from real-world cancer care settings about the implementation and effectiveness of a group-based multimodal tele-prehabilitation program that aims to rapidly engage cancer patients and caregivers to optimize their physical and mental health. This promising relatively low-cost prehabilitation intervention has the potential to accelerate and facilitate adoption of healthy lifestyles and self-management strategy early in the cancer continuum with the objective to optimize the whole cancer care experience. By assessing the implementation and effectiveness in various clinical contexts, this project will provide useful knowledge for scaling-up of the intervention across Quebec. Ultimately, embedding multimodal prehabilitation as a standard cancer care has the potential to accelerate patient and caregiver management, facilitate follow-up, improve health outcomes and reduce the burden on the healthcare system.

## Ethics and dissemination

This multicentre study involves human participants and received ethic approval by the CHUM Research Ethics Board (no. MP-02-2024-11827); CHUS and CIUSSS du Saguenay-Lac-St-Jean provided ethic institutional convenience. Participation is voluntary, and all participants (patients, caregivers, health professionals and managers) will provide written informed consent prior to participation.

Our team will disseminate coACTIF implementation and effectiveness results through reports to stakeholders - including participants, QEP, healthcare professionals and community networks, scientific manuscripts and presentation at clinical and scientific conferences. coACTIF is cast as an integrated Knowledge Transfer and Exchange (iKTE) strategy that aims to promote the reciprocal exchange of knowledge between researchers, students, health professionals and managers, and especially cancer patients and caregivers who are central actors in cancer care. iKTE deliverables will be co-constructed and vetted by multiple stakeholders to increase equity in access to scientific knowledge and to contribute to citizen scientific literacy. iKTE deliverables include: i) the educational material available on coACTIF platform, co-created and co-constructed with scientific experts, patients and caregivers, ii) comics, to share key information to patients and caregivers using few words and attractive pictures. This innovative medium encourages empathy and reinforce comprehension and durable recall that helps convey messages easily and quickly, making knowledge accessible for people with low literacy, iii) scientific articles published in peer-review journals, iv) brief reports and webinars to disseminate findings in an accessible format to patients, caregivers, the general public, stakeholders and program managers, v) communications at clinical practice congress and scientific conferences and iv) open science knowledge mobilization workshops and public events in each region involved in the project: Montréal, Sherbrooke, Chicoutimi with key stakeholders, health professionals and the public.
