# Supplementary material for: Multimodal group-based tele-prehabilitation for cancer patients and caregivers: a pragmatic multicentre hybrid implementation-effectiveness study protocol
Source: Front Oncol. 2025 Sep 26;15:1566489. doi: 10.3389/fonc.2025.1566489 (PMC12510851; doi:10.3389/fonc.2025.1566489)
Supplement: Supplementary file 1 [file DataSheet1.pdf]

Supplementary material

The PRagmatic-Explanatory Continuum Indicator Summary 2 (PRECIS-2) wheel for coACTIF study

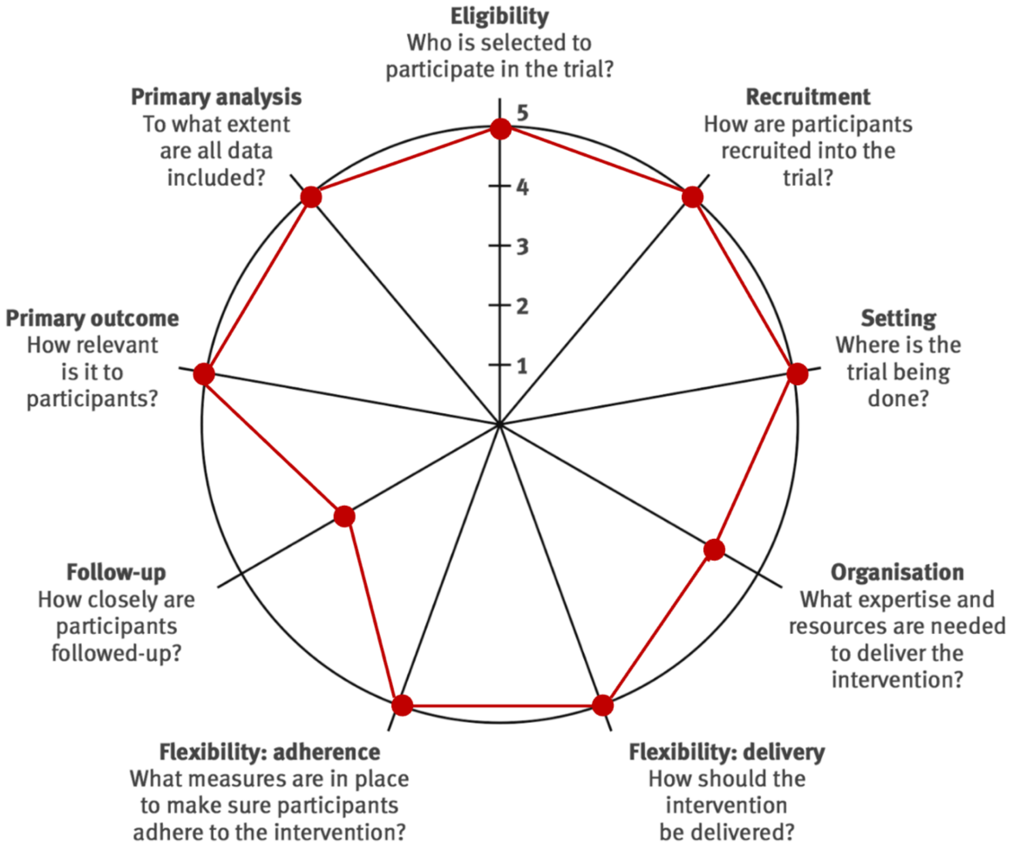

© coACTIF – PRECIS-2 Wheel

Exercise Session Structure

Exercise session with interval aerobic stations runs on Monday and Friday while exercise session with continuous aerobic stations runs on Wednesday.

Exercise session structure with INTERVAL aerobic stations

STATIONS	SETS	BORG-10 scale	EFFORT DURATION	REST TIME (between exercises)
**WARM-UP – 10 min**				
Mobility exercises	-		5 min	-
Cardio (low intensity)	-		5 min	-
**MAIN SESSION – 45 min**				
**A – Aerobic –> Intervals**[a]				
High knees	5	5-6	30sec	-
		2-3	30 sec	-
**B – Resistance**[b]				
Surgery-specific	2	3-6	45 sec	30 sec
Upper body – Pull			45 sec	30 sec
Lower body – Squat variations			45 sec	30 sec
Upper body – Push			45 sec	30 sec
**C – Aerobic –> Intervals**[a]				
Speed skaters	5	5-6	30 sec	-
		2-3	30 sec	-
**D – Resistance**[b]				
Abdominals		3-6	45 sec	30 sec
Glutes	2		45 sec	30 sec
Lower back			45 sec	30 sec
**E – Aerobic –> Intervals**[a]				
Quick feet (2 feet in, 2 feet out)	5	5-6	30 sec	-
		2-3	30 sec	-
**COOL-DOWN – 5 min**				
Static stretches and breathing	-	1-2	5 min	-

[a]Complete the 5-minute aerobic station using 30-second intervals. We recommend varying the exercises every minute, either by alternating between two exercises or by performing five different exercises. Be sure to move in all four directions throughout the session (front, back, right side, left side). Examples are provided in Table 1.

[b]Complete the resistance exercises as a circuit. Take a maximum 2-minute break at the end, then repeat the circuit. Use this break to adjust or correct exercises if needed.

© coACTIF – Exercise Session Structure
June 10, 2025

Exercise session structure with CONTINUOUS aerobic stations

STATIONS	SETS	BORG-10 scale	EFFORT DURATION	REST TIME (between exercises)
**WARM-UP – 10 min**				
Mobility exercises	-	2-3	5 min	-
Cardio – Low intensity	-		5 min	
**MAIN SESSION – 45 min**				
**A – Aerobic –> Continuous**[a]				
Jumping jacks (with or without jump)	1	3-4	1 min	-
High knees			1 min	
Shadow boxing			1 min	
Butt kicks			1 min	
Quick feet (2 feet in, 2 feet out)			1 min	
**B – Resistance**[b]				
Surgery-specific	2	3-6	45 sec	30 sec
Upper body – Pull			45 sec	30 sec
Lower body – Squat variations			45 sec	30 sec
Upper body – Push			45 sec	30 sec
**C – Aerobic –> Continuous**[a]				
Speed skaters	1	3-4	1 min	-
Quick feet			1 min	
Speed skaters			1 min	
Quick feet			1 min	
Speed skaters			1 min	
**D – Resistance**[b]				
Abdominals	2	3-6	45 sec	30 sec
Glutes			45 sec	30 sec
Lower back			45 sec	30 sec
**E – Aerobic –> Continuous**[a]				
Jumping jacks (with or without jump)	1	3-4	1 min	-
High knees			1 min	
Shadow boxing			1 min	
Butt kicks			1 min	
Quick feet (2 feet in, 2 feet out)			1 min	
**COOL-DOWN – 5 min**				
Static stretches and breathing	-	1-2	5 min	-

[a]Complete the 5-minute aerobic station at a continuous intensity. We recommend varying the exercises every minute, either by alternating between two exercises or by performing five different exercises. Be sure to move in all four directions throughout the session (front, back, right side, left side). Examples are provided in Table 2.

[b]Complete the resistance exercises in a circuit. Take a maximum 2-minute break at the end, then repeat the circuit. Use this break to adjust or correct the exercises if needed.

Fidelity checklist

Fidelity checks are conducted to ensure that the intervention provided aligns with the protocol. Observation visits will be conducted for a total of 10% of sessions. The observer attends virtual sessions with their camera off, then provides comments on the structure and flow of the session as well as on social interactions. If any elements deviate from the protocol, the observer must provide details on the situation and propose a strategy to address it.

Date :
Observer's First and Last Name:
QEPs' First and Last Names:
Session number :
Fidelity check number :

Fidelity Check	YES	NO	Comments
**1 – Overall Session**			
**Lead QEP...**			
– Provides clear and concise instructions and demonstrations (breathing, positioning, etc.)			
– Explains the purpose of the exercises (or station) to the participants			
– Corrects participants' form to ensure safety			
– Adjusts exercises as needed based on participants' health conditions			
– Checks participants' energy levels during the session			
– Uses a reassuring, positive, and dynamic voice			
– Meets coACTIF protocol expectations (delivers the program safely, effectively, and competently)			
– Specific comments for the lead kinesiologist (overall session flow, specific points to raise, positive observations, etc.)			
**Moderator QEP...**			
– takes attendance at the beginning of the session (alone, at home, absentees)			
– Renames participants appropriately if needed			
– Puts both QEPs in spotlight mode for all participants			
– Ensures participant safety through observation and necessary corrections (corrects form, addresses concerns, ensures participants are always "on camera")			
– Uses a reassuring, positive, and dynamic voice			
– Plays an active role during the session (demonstrates variations, guides participants, etc.)			
– Meets coACTIF protocol expectations (delivers the program safely, effectively, and competently)			
– Specific comments for the moderator (overall session flow, specific points to raise, positive observations, etc.)			
**Session management**			
– Both QEPs divide responsibilities effectively			
– Music is shared with participants during effort phases (specify who shared it)			
– The session takes place in a positive environment that promotes motivation			
– If any situation occurs (e.g., a participant disappears from the screen), both kinesiologists respond as a team and manage the situation according to the adverse event protocol or in an appropriate manner			

**2 - Session structure**			
**Warm-up**			
– Includes 5 minutes of low-intensity cardio			
– Includes mobility exercises targeting major joints			
**Main session**			
– 5 min cardio completed			
– Strength training block 1 completed			
– 5 min cardio completed			
– Strength training block 2 completed			
– 5 min cardio completed			
**Cool-down**			
– 2 upper body stretches			
– 2 lower body stretches			
– 1–2 minutes of deep breathing			
**Overall structure**			
– Targeted intensity levels were appropriate			
– Duration of effort phases was appropriate			
– Overall session structure was respected			
**3 – Social interactions**			
**Between QEPs**			
– Communication between the 2 kinesiologists is always effective and professional			
**Between participants**			
– Before the session			
– During the session			
– After the session			
– Participants communicate with each other respectfully			
**Between QEPs and participants**			
– Before the session			
– During the session			
– After the session			
– Communication between both parties is respectful			
– QEPs foster an environment in favor of interactions and social support			
**4 – General comments**			
– Any additional comments :			
